# Preface: Franz-Josef Kaup and the development of the Pathology Unit at the German Primate Center

**DOI:** 10.5194/pb-4-229-2017

**Published:** 2017-10-27

**Authors:** Kerstin Mätz-Rensing, Martina Bleyer

**Affiliations:** German Primate Center, 37077 Göttingen, Germany

## Abstract

This special issue about selected diseases of nonhuman primates was created in honor of Franz-Josef Kaup, who worked as a primate pathologist at the German Primate Center (DPZ) for 25 years. In 1992, Franz-Josef Kaup started his career at the DPZ as head of the working group Experimental Pathology. Prior to that he worked as a research assistant in the division Electron Microscopy at the Institute of Pathology of the University of Veterinary Medicine in Hanover. He was very experienced in the field of electron microscopy and used this expertise to establish a central electron microscopy laboratory at the DPZ. In the beginning, research of the working group Experimental Pathology was focused on gastrointestinal and respiratory infections and was closely related to projects of the Department of Virology. At that time, experimental infections of rhesus macaques with simian immunodeficiency virus (SIV) and associated opportunistic infections became the main subject of his research. The contribution of Christiane Stahl-Hennig and coauthors about SIV-induced cardiovascular diseases reflects the still ongoing collaboration in this research field. After merging the Experimental Pathology and Primate Husbandry in 1996, Franz-Josef Kaup headed the newly created Department of Veterinary Medicine and Primate Husbandry. This department became the central service unit of the DPZ in 1999 and offered a broad spectrum of services in veterinary diagnostics, primate husbandry, and animal welfare, which was intensively used by many internal and external scientists. In 2001, Walter Bodemer joined the group and the scientific contents expanded with a new focus on the pathogenesis of prion diseases. Some important aspects of this era are summarized in the work of Walter Bodemer.

Several animal models were successfully established under the leadership of Franz-Josef Kaup, including an animal model for *Helicobacter pylori* infections. Currently, the main research focus is on the pathogenesis of orthopoxvirus infections. Kerstin Mätz-Rensing and colleagues established a new animal model for orthopox virus infections, which is based on a natural outbreak of the disease in a private New World monkey husbandry. During this outbreak, a new orthopox virus was discovered, which was named calpox after its host species. This animal model was used for pathogenetic and vaccination studies in orthopox virus research. Over the years, a close cooperation with several working groups working in this important field of virus research arose. The contribution of Kerstin Mätz-Rensing gives an example of the experimental work with the newly discovered calpox virus in close collaboration with colleagues at the Robert Koch Institute in Berlin.

The Department of Veterinary Medicine and Primate Husbandry was reorganized into the Pathology Unit and the Cost Center Primate Husbandry, both headed by Franz-Josef Kaup. Since 2004, the Pathology Unit has been composed of the Primate Pathology Group headed by Kerstin Mätz-Rensing, the Dermatopathology Group headed by Bärbel Löblich-Beardi and the Herpes Virus Group headed by Dieter Jentsch. The research spectrum broadened with the dermatopathology- and herpes-virus-related research. As head of the Primate Husbandry Franz-Josef Kaup focused on the herpes B problematic in macaques and supported the establishment of herpes B diagnostics at the DPZ. Herpes B is of special interest when handling macaques in research projects and breeding colonies. The development of a valuable herpes B screening test and the current state of art at the DPZ is described in the contribution of Stefan Pöhlmann and colleagues.

Another important disease that can cause health problems in breeding colonies is endometriosis. The contributions of Eva Gruber-Dujardin and Ivanela Kondova address this disease and its related research and lead over to spontaneous diseases of nonhuman primates, which have been in the focus of the Pathology Group for many years.

Over the years, several important cases of emerging diseases, rarely occurring neoplasia, and unique disease entities in nonhuman primates were described by this group. The work by Karen Lampe about a unique T-cell lymphoma in a patas monkey and by Nicole Cichon about a unique granulomatous arteritis are examples of those spontaneous diseases observed during routine pathomorphologic diagnostics. The opportunity to work on those extraordinary cases emerged from close contact with several German zoos that sought the expertise of Franz-Josef Kaup's group in the field of primate diseases and pathology. Other interesting case reports resulted from the routine clinical and diagnostic work such as the contribution of Tamara Becker about Guillain–Barré syndrome in a rhesus monkey and Roland Plesker, a close colleague from Paul Ehrlich Institute in Frankfurt, about a gallstone problematic. Over the years, Franz-Josef Kaup supported the work of colleagues from the Department of Behavioral Ecology and Sociobiology and other colleagues predominantly working in the field in the natural habitats of nonhuman primates. One of the biggest health problems among natural primate colonies is parasites, and the articles by Andrea Springer, Peter Kappeler, Fabian Leendertz reflect this situation.

A cooperation with the working group of the Fraunhofer Institute for Toxicology and Experimental Medicine (ITEM) in Hanover headed by Franziska Dahlmann was established in 2010. The ITEM group worked on primate models for obstructive lung diseases and asthma and was supported by the working group Anatomy/Pathology of the Respiratory Tract headed by Martina Bleyer. The contributions of Martina Bleyer and Franziska Dahlmann in this issue will highlight aspects of their work. Over the years, Franz-Josef Kaup's main research focuses were lung diseases, starting with the work in Hanover on chronic obstructive pulmonary disease in horses. With the new project on an asthma model in nonhuman primates, the circle of his lifework is complete.

